# Hybrid laparoscopic repair of complex abdominal wall hernias with transabdominal partially extraperitoneal mesh fixation: preliminary results

**DOI:** 10.3389/fsurg.2025.1575403

**Published:** 2025-06-18

**Authors:** Sarah Mahmood, Yusuf Moollan, Sarit S. Badiani, Christophe R. Berney

**Affiliations:** ^1^ Department of Surgery, Bankstown-Lidcombe Hospital, Sydney, NSW, Australia; ^2^ Faculty of Medicine, University of New South Wales, Sydney, NSW, Australia

**Keywords:** hybrid hernia repair, ventral hernia, tape, incisional hernia, mesh, quality of life

## Abstract

**Background:**

There are two conventional approaches to abdominal wall hernia repairs that aim to achieve anatomical restoration. Open approaches have the advantage of complete hernial sac excision with freedom of mesh placement. In comparison, the advantages of the laparoscopic approach involve shorter hospital stays, less postoperative pain, and fewer postoperative complications. More recently, the hybrid approach, which combines the two techniques, has gained popularity as a way to potentially reap the benefits of both. Our aim was to determine whether this hybrid approach can achieve the same benefits, irrespective of hernia size, age, and body mass index (BMI). Primary outcome of interest was hernia recurrence. Secondary outcomes included postoperative complications, pain, and quality of life (QoL).

**Method:**

Medical records of all patients who underwent hybrid laparoscopic hernia repair (HLHR) with transabdominal partially extraperitoneal (TAPE) mesh fixation between 2017 and 2023 were retrieved from a prospectively maintained institutional database and retrospectively analyzed. Intra- and postoperative complications, as well as hernia recurrence, were recorded. QoL was assessed using the Carolinas Comfort Scale (CCS).

**Results:**

There were 37 patients (21 women, 56.8%) with a mean age of 66 years and BMI of 34.2 kg/m^2^ who underwent hybrid hernia repair. Of them, 34 (91.9%) were incisional hernias, of which 50% were recurrent. Mean hernia defect size was 96.8 cm^2^. Of the patients, 12 (32.4%) received preoperative chemical component separation with botulinum toxin A (BTA); this group had a significantly higher BMI and hernia size compared to the no BTA group (37.7 vs. 32.5; *p* = 0.048; 174.5 cm^2^ vs. 59.5 cm^2^; *p* = 0.0002). There were no intraoperative complications; however, there were 8 (21.6%) minor postoperative complications. After a mean follow-up of 40 months, we recorded one hernia recurrence at 23 months postoperatively (2.7%). In addition, out of 29 (78.4%) patients assessed for QoL, the median and mean scores were reported as 0 out of 115 and 2.6 out of 115 points scale, respectively.

**Conclusions:**

HLHR with TAPE mesh fixation is safe, with satisfactory mid- to long-term outcomes, irrespective of hernia size and BMI.

## Introduction

Abdominal wall defects are common surgical pathologies. Primary ventral hernias account for approximately 20% of the adult population, while incisional hernias develop in up to 30% of patients after midline laparotomy (1). The two conventional approaches to ventral/incisional hernia repair to achieve anatomical restoration of the abdominal wall are either via the open or minimally invasive approach, each with its own advantages and disadvantages. The advantages of laparoscopic hernia repair (LHR) include shorter hospital stay, less postoperative pain, increased patient satisfaction, and fewer overall postoperative complications, whereas the disadvantages include the technical learning curve, limited hernia size (generally <10 cm in size), higher rates of seroma formation in the retained hernia sac with poor cosmesis, and longer operation time (2). In comparison, open hernia repair (OHR) allows the benefit of complete hernial sac excision and more freedom with mesh placement such as retromuscular, extraperitoneal, or onlay approach. However, it is associated with longer hospital stay, increased postoperative pain, and increased risk of complications. To date, there is no conclusive evidence to suggest one technique is superior to the other when it comes to hernia recurrence rates, except for smaller fascial defects in the range of 2–6 cm, as suggested in a recent large nationwide database study (3). Nevertheless, both techniques are considered safe depending on the surgeon’s experience.

The hybrid procedure combines the laparoscopic and open hernia approaches and has gained popularity in the field of abdominal wall reconstruction. This technique endeavors to overcome the disadvantages associated with both procedures while retaining their respective advantages. The approach typically begins with laparoscopic adhesiolysis and complete reduction of the hernia contents, followed by peritoneal stripping around the defect. This is then complemented by open transcutaneous excision of the residual subcutaneous hernia sac and primary fascial closure. Pneumoperitoneum is then re-established, and a laparoscopic transabdominal partially extraperitoneal repair (TAPE) is performed (4). A recent systematic review and meta-analysis comparing purely laparoscopic with hybrid laparoscopic hernia repair (HLHR) found that postoperative complications seemed to be less frequent with the hybrid approach (5).

The aim of this retrospective study was to evaluate whether the hybrid approach to hernia repair offers comparable outcomes to standard laparoscopic repair, regardless of hernia size, patient age, or body mass index (BMI), with particular focus on complication rates and mid- to long-term recurrence.

## Method

This was a retrospective cohort study of all patients who underwent elective hybrid repair of primary ventral, incisional, or recurrent hernias, performed at two institutions under the care of one of the coauthors (CB) between January 2017 and May 2023. The selection of patients undergoing hybrid repair was primarily based on having a BMI > 25 and a large hernia defect >4 cm. The electronic medical records were specifically screened for patient demographics such as age, gender, and number and size of hernia defects after obtaining informed consent from the patients. Hernia location was determined according to the European Hernia Society (EHS) incisional hernia classification (6). All patients had their regular postoperative review at 2 weeks, 6 weeks, 3 months, and 6 months to assess for recurrence and other postoperative complications. They were subsequently discharged back to the community under the care of their general practitioner if no complications were identified during follow-up, with a notification to come back for further review if deemed necessary. Telehealth follow-up was performed in 2024 to assess subjective symptoms and quality of life (QoL) after hernia repair.

The primary outcome was hernia recurrence. This was assessed at the 6-month in-person follow-up and self-reported by patients during the telehealth follow-up; thus, subclinical recurrences may be underreported. Secondary outcomes included postoperative complications, postoperative pain, and QoL measures as determined by the Carolinas Comfort Scale (CCS) (7). This scale assesses the patient’s perception of various postoperative outcomes, including sensation of mesh, pain, and limitations to daily activities (Supplementary Appendix 1). This study was approved by the local Institutional Review Board and performed in accordance with the Declaration of Helsinki.

### Surgical technique

Patients were started on a preoperative high-protein and low-calorie diet, depending on their respective BMI. Preoperative chemical component separation with botulinum toxin A (BTA) injections administered under ultrasound guidance to the lateral abdominal wall was offered to aid primary fascial closure. The decision to administer BTA was based on the surgeon’s discretion and dependent on the patient’s BMI, size of the hernial defect, and previous hernia recurrences. BTA was administered 2–4 weeks before the hernia repair, using 500 U of Dysport® (Ipsen Pty Ltd.) injected equally into both the external and internal oblique muscles at three levels on each side, totaling 12 injection sites (approximately 41.6 U per site).

A thorough anesthetic assessment was completed preoperatively as per standard protocol and the hernia defect was marked on the day of surgery. Prophylactic intravenous antibiotics and deep vein thrombosis prophylaxis with subcutaneous enoxaparin were administered before induction. The patient was positioned supine on the operating table and a WHO surgical safety checklist time-out was completed. General anesthesia was induced, calf compressors were applied, and an indwelling catheter (IDC) was inserted. Under sterile conditions, pneumoperitoneum was established either via Verress needle entry at Palmer’s point in the left upper quadrant or through an open infraumbilical Hasson technique if the hernia defect was lateral. After establishing pneumoperitoneum, a 10 mm 30° scope and two 5 mm laparoscopic ports were inserted on the left side of the abdomen along the anterior axillary line (for midline defects) to facilitate adhesiolysis and reduction of hernia contents. One or two additional 5 mm ports were placed on the contralateral side as needed.

**Figure 1:**
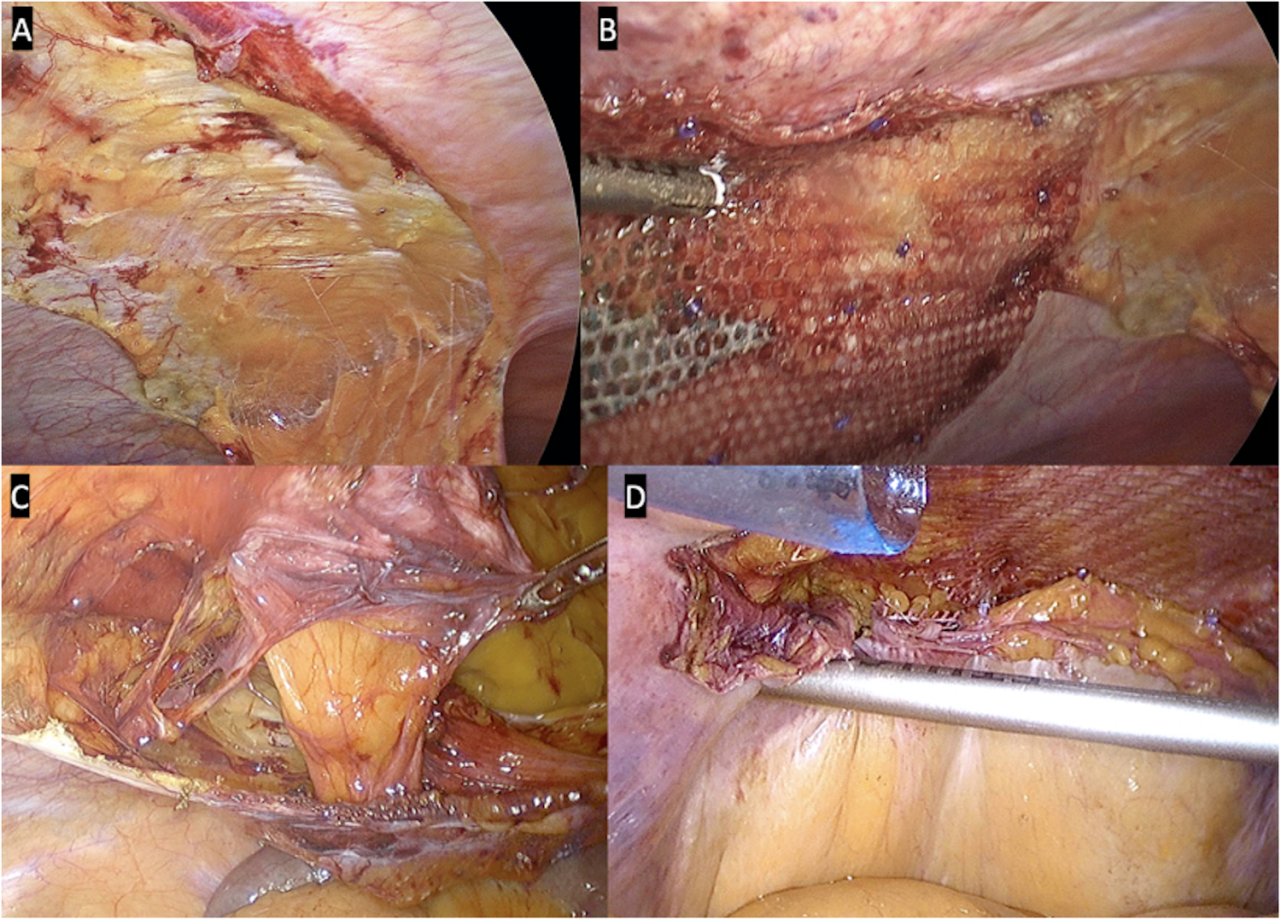
Laparoscopic views of peritoneal flaps. **(A)** Dissected falciform ligament. **(B)** Transabdominal partially extraperitoneal (TAPE) repair. **(C)** Medial ligaments taken down showing hernia defect. **(D)** Partial closure of distal peritoneum (TAPE).

The hernia defect was identified and careful adhesiolysis was performed using a combination of sharp, electrocautery (hook), and ultrasonic energy devices (Harmonic scalpel, Ethicon Endo-Surgery, Inc.; Thunderbeat, Olympus Medical Systems Corporation). The entire hernia contents, including viscera and omentum, were safely reduced into the peritoneal cavity, leaving only the subcutaneous sac within the defect. A wide peritoneum flap was then developed around the defect. In midline hernias, this typically involved division of the falciform ligament proximally and both medial ligaments distally. At least 5 cm of parietal peritoneum surrounding the hernia defect was stripped to enable secure placement of the mesh directly onto the posterior muscle fascia with adequate overlap (Figure 1).

**Figure 2:**
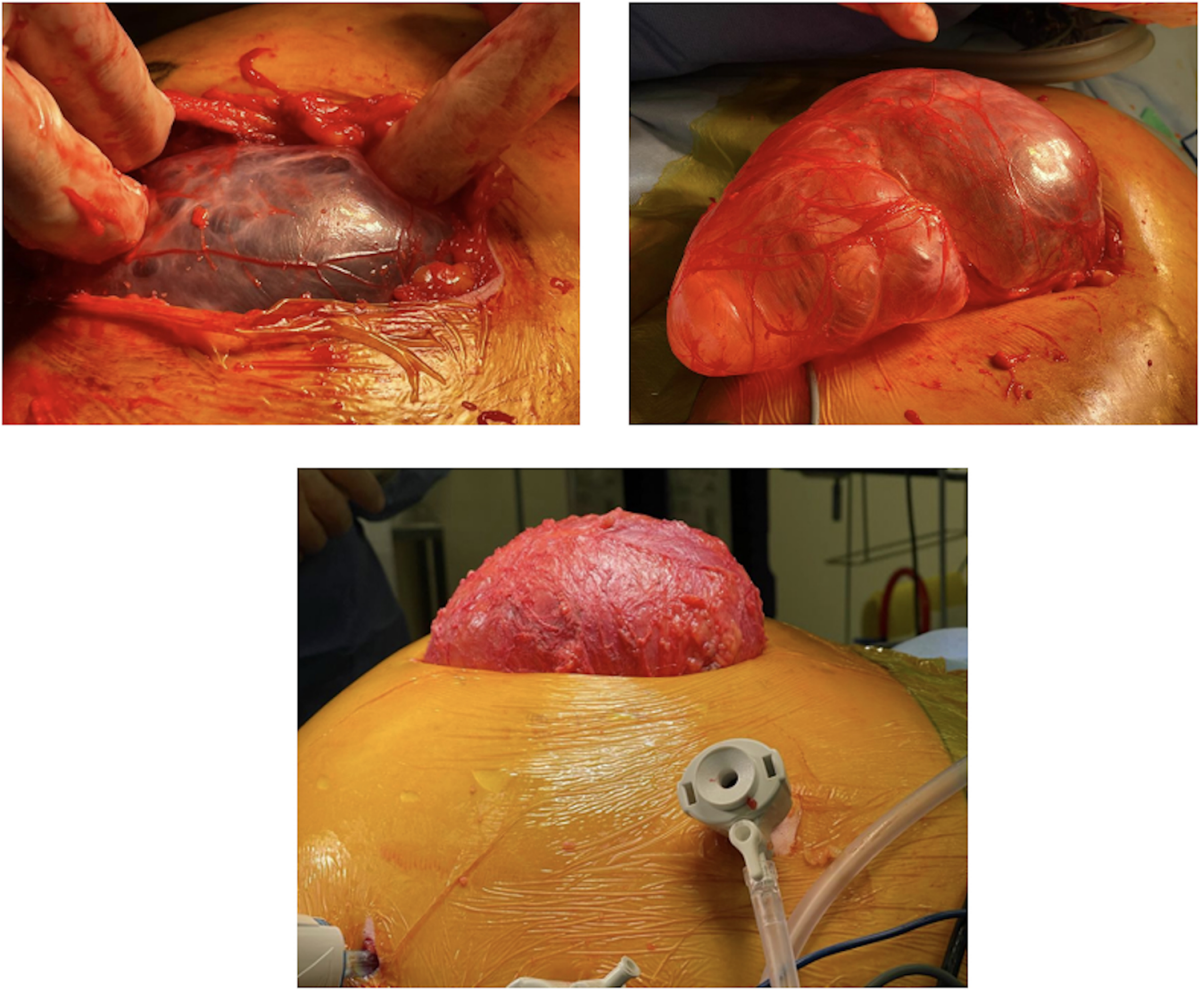
Intraoperative images demonstrating dissected subcutaneous ventral hernia sac under continuous pneumoperitoneum guidance.

Under continuous pneumoperitoneum guidance, a tailored skin incision was made to facilitate dissection of the remanent subcutaneous hernia sac down to the fascial edges (Figure 2). The pneumoperitoneum was temporarily released, the hernia sac entirely excised, and the fascia was primarily closed using a combination of continuous and interrupted size 1 polydioxanone sutures (PDS® II, Ethicon Endo-Surgery, Inc., CA).

Pneumoperitoneum was re-established at 8–9 mmHg and an appropriately sized synthetic mesh was inserted laparoscopically into the extraperitoneal space. The mesh was positioned and secured to the posterior fascia using a minimum of five extracorporeal 2/0 polydioxanone sutures (PDS® II, Ethicon Endo-Surgery). One suture was placed centrally to correspond with the midpoint of the hernia repair, using an Endo Close™ needle (Medtronic, Minneapolis, MN) and reinforced with SecureStrap™ (Ethicon™ Inc., San Angelo, TX). For mesh fixation in lateral abdominal wall defects, the pelvis, or subxiphoid region, where stapling may be unsafe, fibrin sealant (Tisseel™, Baxter International Inc., Deerfield, IL) was used. When feasible, the mobilized parietal peritoneum was reattached to the posterior abdominal wall, covering part of the prosthetic mesh. After confirming hemostasis, the greater omentum was repositioned on the surface of the bowel to provide an additional protective barrier. Ports were removed under vision, port sites were infiltrated with local anesthetic, and closed with 3/0 Monocryl (poliglecaprone 25, Ethicon™). No intra-abdominal drain was inserted. Postoperatively, patients wore an abdominal binder continuously (24/7) for a minimum of 2 weeks, then during the day for another 6–8 weeks (or longer after Botox administration). The IDC was removed within 24–48 h.

## Results

Of the 40 patients who underwent HLHR, three were lost to follow-up and therefore excluded from the study. The mean age of the remaining 37 patients was 66 years, with a median BMI of 34.2 kg/m^2^ (range: 25–56). The male-to-female ratio was 0.76 (16 and 21 patients, respectively). A total of 34/37 (91.9%) patients had incisional hernias, of which 17 (50%) were referred for recurrent ventral/incisional hernia (Table 1). The size of the hernia defects varied widely, in the range of 12–350 cm^2^, with a mean of 96.8 cm^2^. Approximately half of those hernias were centered in the umbilical region (M3, 51.4%). Multiple fascial defects were observed in 11 (29.7%) patients. Preoperative chemical component separation with BTA was administered in 12/37 (32.4%) cases. Patients who received preoperative BTA had a significantly higher BMI compared to those who did not (37.7 vs. 32.5; *p* < 0.05). In addition, the mean hernia size in the BTA group was significantly larger than in the non-BTA group (174.5 cm^2^ vs. 59.5 cm^2^; *p* < 0.001).

**Table 1:** Characteristics of patients undergoing hybrid hernia repair.

Age, years (range)	66 (29–88)	
Male/female	16/21	43.2%/56.8%
ASA classification	I	0
	II	17
	III	14
	IV	6
European Classification of Incisional Hernias:	M1	0%
M1 subxyphoidal, M2 epigastric, M3 umbilical, M4 infraumbilical, M5 suprapubic, L1 subcostal, L2 flank, L3 iliac, L4 lumbar	M2	10.8%
	M3	51.4%
	M4	10.8%
	M5	8.1%
	L1	0%
	L2	10.8%
	L3	8.1%
	L4	0%
Hernia type	Primary	8.1%
	Incisional	91.9%
	Recurrent	50%
Preoperative BTA injection	12/37	32.4%
Mean hernia size, cm^2^ (range)	96.8 (12–350)	*P* < 0.001
Preop. BTA	174.5 (48–350)	
No BTA	59.5 (12–160)	
Mean BMI, Kg/m^2^ (range)	34.2 (25–56)	*P* < 0.05
Preop. BTA	37.7 (25–53.8)	
No BTA	32.5 (25–56)	
Mesh Type (number)	Symbotex (18)	41.9%
43 meshes in 37 patients—4 with double and one with triple meshing	Ventralight ST (8)	18.6%
	Ventralex ST (3)	7%
	Sepramesh (3)	7%
	Parietex -3D (3)	7%
	Anatomical (2)	4.6%
	Polypropylene (3)	7%
	Phasix (2)	4.6%
	Physiomesh (1)	2.3%

A total of 43 meshes were used across the sites, with four patients receiving two prostheses and patient receiving three. The types of prostheses used are summarized in Table 1. The most commonly used mesh was Symbotex™ Composite (Medtronic), accounting for 41.9% of cases, followed by Ventralight™ ST (Bard®, Franklin Lakes, NJ) in 18.6%. The average mesh size was 289.2 cm^2^. When comparing patients who received preoperative BTA to those who did not, the mean mesh sizes were 379.8 and 243.9 cm^2^, respectively (*p* = 0.01). Seven meshes were secured onlay, three extraperitoneally and 33 via a partially extraperitoneal (TAPE) approach. Mesh fixation primarily utilized SecureStraps™ in 34 (91.9%) cases. Fibrin sealant alone was used in two patients with lateral defects, and sutures only were used in one high-risk patient aged 74 years (ASA IV) with a BMI of 53.8 kg/m^2^ and the largest recurrent incisional hernia defect in the series (350 cm^2^). In this case, a 30 × 30 cm Parietex hydrophilic 3D mesh was fixed onlay. Fibrin sealant was also used for mesh fixation in three patients with concurrent inguinal hernias.

**Figure 3:**
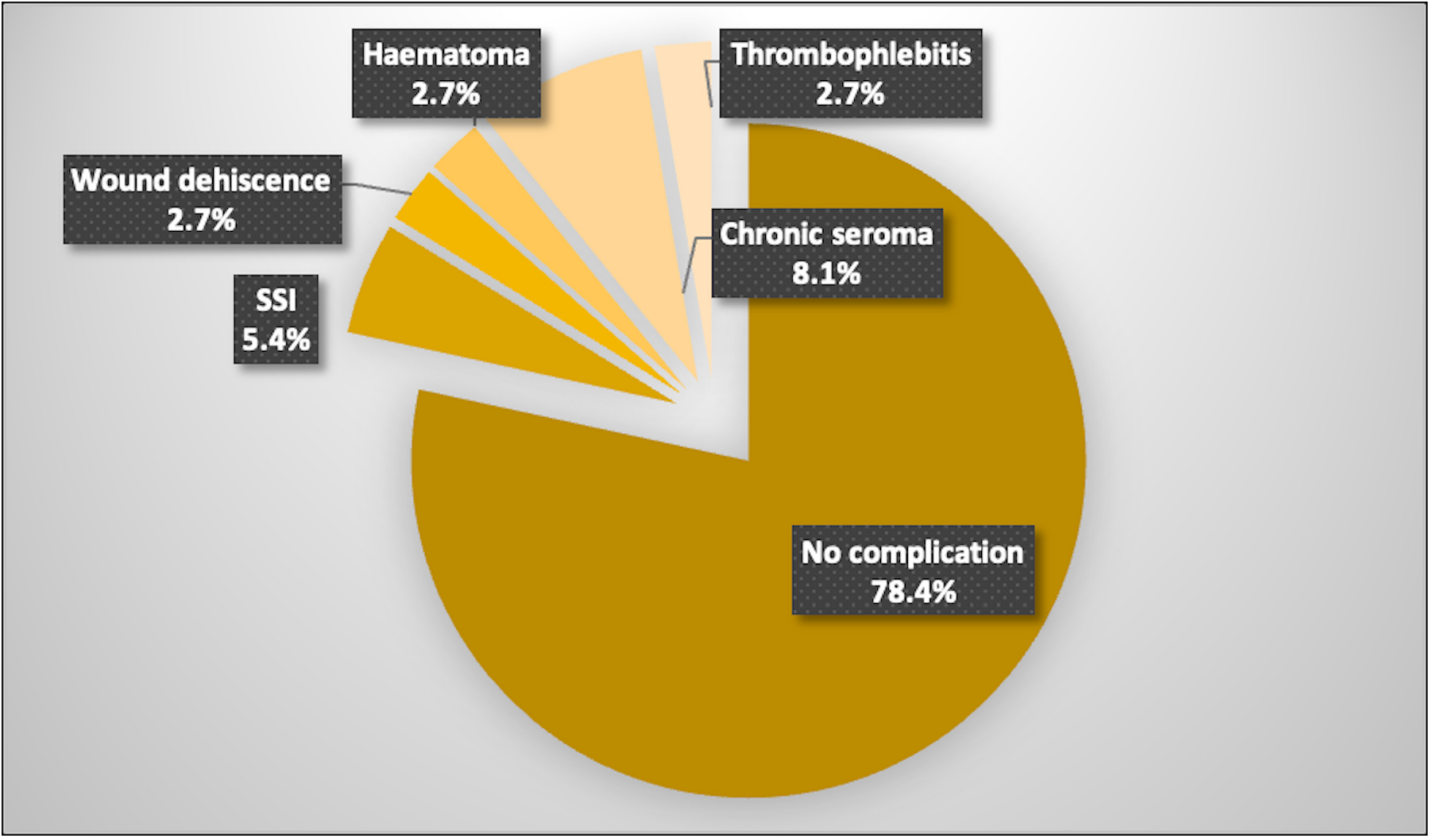
Distribution of postoperative complications in patients undergoing hybrid laparoscopic hernia repair.

No intraoperative complications were recorded and no anterior or posterior component separation was necessary. There were eight (21.6%) minor postoperative complications (Figure 3), including superficial site infections (SSI) in two cases requiring oral antibiotics, one wound dehiscence necessitating insertion of a vacuum assisted closure (VAC) dressing, one superficial thrombophlebitis from a canula site, a chronic subcutaneous hematoma requiring drainage on a patient on apixaban, and three chronic seroma formations including one capsule excision at 8 months postoperatively in a morbidly obese patient (BMI: 44.4 kg/m^2^). The mean overall follow-up was 40 months (range: 6–113 months) with combined face-to-face and telehealth interviews postoperatively. The mean face-to-face follow-up was 11.6 months (range: 6–63 months). We identified only one patient, aged 77 years, with a BMI of 35 kg/m^2^ after gastric bypass who had a recurrent incisional hernia that occurred at the 23-month follow-up (overall recurrence rate of 2.7%). Her initial procedure combined mesh repair of an incisional hernia (defect: 150 cm^2^) after BTA injection along with repair of a large divarication and abdominoplasty.

In addition, 29 (78.4%) patients participated in a standardized QoL assessment after hernia repair using the Carolinas Comfort Scale (Supplementary Appendix 1). The median score was 0 out of a possible 115 points. The highest recorded score was 40/115 points, which was mainly associated with pain on movement reported by the only patient in the series who experienced hernia recurrence. The mean score across the cohort was 2.6/115 points.

## Discussion

Abdominal wall hernia repairs are common procedures, with varying recurrence rates reported in the literature—up to 54% when repaired primarily and 36% with mesh reinforcement (8, 9). However, hernia recurrence is multifactorial, with hernia size being a significant risk factor, especially with defects wider than 10 cm (10). In addition, a high BMI (>25 kg/m^2^) is also associated with a higher risk of recurrence, with rates up to 18% compared to 5% in patients with BMI < 25 kg/m^2^ (10). Other risk factors that may influence the hernia recurrence rate include hernia site, smoking, chronic obstructive pulmonary disease, and wound infection.

Using a combined hybrid/laparoscopic approach with a standardized surgical technique for all abdominal wall hernias, we recorded a recurrence rate of just 2.7%, despite a high-risk cohort with an average BMI of 34.2 kg/m^2^ (15 patients with BMI >35 kg/m^2^), and an average defect size of 96.8 cm^2^. A 2020 study by Van den Dop et al., which followed a similar surgical technique, reported comparable results with a low hernia recurrence rate of 5.6% and a seroma rate of 2.8% (11). However, the majority of patients in that study had smaller hernias (<5 cm) and a shorter follow-up time of 1 month. Conversely, a recent randomized control trial by Hiekkaranta et al. compared laparoscopic and hybrid repairs for hernia defects of 2–7 cm in size and found recurrence rates of 20% in both groups after a mean follow-up of 87 months (12). The higher recurrence rates were likely attributed to the use of absorbable tackers for mesh fixation.

One major concern after laparoscopic repair is the formation of chronic seromas due to the presence of a remnant hernial sac. However, our study shows that complete dissection and excision of the subcutaneous sac, followed by primary fascial closure, may significantly reduce the rate of seroma formation and its associated complications, as demonstrated in our observed rate of 8.1%. We also believe that prolonged use of an abdominal binder may potentially minimize seroma formation, but the current evidence in the literature is limited. A randomized multicenter study comparing laparoscopic and hybrid repair approaches reported seroma rates of 12.6% in the hybrid group compared to 31% in the laparoscopic group (13). Our results demonstrate even lower seroma rates at 8.1%.

To date, there is much contention regarding the selection of an appropriate mesh. The various prosthetic mesh options available include synthetic, biologic, and biosynthetic. Data regarding the safety and efficacy of these different mesh types remain inconclusive largely because of surgeon preference. Synthetic mesh remains the most commonly used due to a longer testing profile demonstrating low recurrence rates and low risk of infection (14). These permanent meshes are generally made of polypropylene or polyester, which tend to have greater mechanical strength (14). General guidelines recommend the use of synthetic meshes for clean wounds, with no preference for clean/contaminated wounds (15). A comprehensive meta-analysis of four randomized control trials comparing synthetic versus biologic meshes found a higher risk of hernia recurrence and surgical site infection with biologic mesh, regardless of whether the surgical field was contaminated preoperatively (16). It advised to ensure a mesh overlap of at least 3 cm for open repair of small ventral hernias (1–4 cm), and of ≥5 cm for open repair of hernias >4 cm and for all laparoscopic hernia repairs (15).

BTA injection is an emerging and increasingly popular technique used as an adjunct for complex ventral hernia repair. BTA is a neurotoxin produced by the bacterium *Clostridium botulinum*, which acts selectively on presynaptic cholinergic nerve terminals to block the release of acetylcholine, resulting in temporary muscle paralysis without systemic effects (17). Applying this neurotoxin to the lateral abdominal wall complex helps paralyze and elongate the muscles, allowing for medialization of the rectus muscles and thereby increasing the chance of primary abdominal wall closure (18). This is an important step as routine closure of the fascial defect reduces the risk of hernia recurrence (19). Chemical component separation with BTA under ultrasound guidance for large hernia defects is also an easy non-invasive procedure that has a significant advantage over the more challenging transversus abdominis release (TAR) technique first introduced by Novitsky et al. (20).

In a prospective observational study, Elstner et al. reported their initial results using preoperative BTA in 32 patients. In six (18.8%) cases, they added a limited endoscopic central external oblique release to facilitate closure (21). Another study comparing fascial closure rates and the need for component separation in complex abdominal wall reconstruction, with or without preoperative BTA, found that patients who received BTA were more likely to undergo component separation than those who did not (61% vs. 47%, *p* = 0.042), despite similar average hernia sizes (251 cm^2^ in the BRA group vs. 240 cm^2^ without BTA) and a mean BMI of 31 kg/m^2^ (22). One possible explanation is that in nearly half of the BTA group, approximately one-third of the total visceral volume was located outside the abdominal cavity, indicating severe loss of domain. In contrast, our 12 patients who received preoperative BTA had a higher mean BMI of 37.7 kg/m^2^, but a significantly smaller average hernia size (174.5 cm^2^), and none exhibited signs of severe loss of domain.

Currently, there is no clear consensus regarding the indications for administering BTA before hernia repair. Although some studies report benefits in fascial defects >5 cm, others reserve its use for defects >12 cm (17). The ideal dosage has not yet been established but is in the range of 100–500 IU in the literature, which is typically divided evenly across three injection sites on each side of the midline. Again, there remains no consensus regarding injection sites or depth of infiltration, with some groups utilizing a two-layer approach (external and internal oblique) and others utilizing a three-layer technique (external and internal oblique, transversus abdominus) with similar results (17). BTA is generally administered at least 2 weeks before the planned procedure date, as that is when it starts gaining maximal effect.

Chronic pain after laparoscopic ventral hernia repair is a significant issue that can impair QoL in the long term. Although most studies tend to measure objective outcomes such as hernia recurrence and surgical site infections, subjective measures such as postoperative pain and QoL tend to be overlooked and underreported. Previous studies have described a postoperative pain incidence in the range of 25%–39% (23, 24). There is also variation in the way subjective QoL outcomes are measured, with the most commonly used scoring systems being the EuraHS-QoL and the CCS. In our study, we used the CCS and demonstrated an overall excellent outcome with minimal chronic postoperative pain issues, as confirmed by a median score of 0/115 and a mean score of 2.6/115 points.

The present study has some limitations. First, it is a retrospective study of a prospectively maintained database, which may introduce bias. Second, the sample size is small and was drawn from two centers, with all procedures performed under the care of a single primary surgeon. As such, the patient cohort may not fully represent the general population. However, this also allowed for a standardized technique across the entire cohort study, reducing technical bias and reflecting surgical expertise. A variety of mesh types were utilized during this surgical repair technique, representing an evolution in institutional preference. Although this introduces a potential confounding variable, no correlation was observed between mesh type and complication rates. Another limitation is the restricted face-to-face follow-up beyond 6 months postoperatively. Although telehealth reviews were subsequently performed, they relied solely on patient self-reporting. Thus, our study may have potentially underreported subclinical hernia recurrences that could have been detected on repeat imaging. Finally, because of the retrospective nature of the study, no comparison groups were included. Therefore, while the hybrid approach appears safe and may offer certain advantages over traditional laparoscopic repair, the current data do not allow for conclusions regarding its superiority or equivalence to existing techniques.

Although all patients had scheduled follow-ups, the study could be further improved with a more structured clinical review with routine implementation of validated QoL scores, such as the Carolinas Scoring System at each visit, and subsequently on a yearly basis with repeat imaging. That being said, patients’ long-term compliance is and will always remain difficult to achieve, and most studies do not implement routine validated QoL assessment tools as part of their follow-up, as it may also be influenced by recall bias. Finally, our study would have also theoretically benefited from standardized mesh choice to minimize confounding variables.

In conclusion, this study demonstrates that the hybrid approach for complex medium- to large-sized abdominal wall hernias is a viable alternative to traditional open and laparoscopic techniques, offering the combined benefits of both without increasing risk. It is associated with low mid- to long-term recurrence rates and a low overall incidence of postoperative complications. Our findings support the use of preoperative BTA injection as a safe, non-invasive approach to facilitate primary fascial closure. We believe it should be more widely available within the general surgical community, as it may reduce the need for more invasive component separation techniques.

## Data Availability

The raw data supporting the conclusions of this article will be made available by the authors, without undue reservation.
