# Supplementary material for: Hybrid laparoscopic repair of complex abdominal wall hernias with transabdominal partially extraperitoneal mesh fixation: preliminary results
Source: Front Surg. 2025 Jun 18;12:1575403. doi: 10.3389/fsurg.2025.1575403 (PMC12213775; doi:10.3389/fsurg.2025.1575403)
Supplement: Supplementary file 1 [file Supplementaryfile1.docx]

The Carolinas Comfort Scale

| Number | Question | Scores |
| --- | --- | --- |
| 1 | Whilst laying down, do you have |  |
|  | Sensation of mesh | 0 1 2 3 4 5 N/A |
|  | Pain | 0 1 2 3 4 5 N/A |
| 2 | Whilst bending over, do you have |  |
|  | Sensation of mesh | 0 1 2 3 4 5 N/A |
|  | Pain | 0 1 2 3 4 5 N/A |
|  | Movement Limitations | 0 1 2 3 4 5 N/A |
| 3 | Whilst sitting up, do you have |  |
|  | Sensation of mesh | 0 1 2 3 4 5 N/A |
|  | Pain | 0 1 2 3 4 5 N/A |
|  | Movement Limitations | 0 1 2 3 4 5 N/A |
| 4 | Whilst performing activities of daily living (getting out of bed, bathing, getting dressed), do you have |  |
|  | Sensation of mesh | 0 1 2 3 4 5 N/A |
|  | Pain | 0 1 2 3 4 5 N/A |
|  | Movement Limitations | 0 1 2 3 4 5 N/A |
| 5 | When coughing, sneezing, or deep breathing, do you have |  |
|  | Sensation of mesh | 0 1 2 3 4 5 N/A |
|  | Pain | 0 1 2 3 4 5 N/A |
|  | Movement Limitations | 0 1 2 3 4 5 N/A |
| 6 | When walking or standing, do you have |  |
|  | Sensation of mesh | 0 1 2 3 4 5 N/A |
|  | Pain | 0 1 2 3 4 5 N/A |
|  | Movement Limitations | 0 1 2 3 4 5 N/A |
| 7 | When walking up or down stairs, do you have |  |
|  | Sensation of mesh | 0 1 2 3 4 5 N/A |
|  | Pain | 0 1 2 3 4 5 N/A |
|  | Movement Limitations | 0 1 2 3 4 5 N/A |
| 8 | When exercising (other than work-related), do you have |  |
|  | Sensation of mesh | 0 1 2 3 4 5 N/A |
|  | Pain | 0 1 2 3 4 5 N/A |
|  | Movement Limitations | 0 1 2 3 4 5 N/A |

Patients reported scores of 0 for no pain or movement limitations, up to 5 for worst symptoms, or N/A if not applicable
